# Cancer Data and Aboriginal Disparities (CanDAD)—developing an Advanced Cancer Data System for Aboriginal people in South Australia: a mixed methods research protocol

**DOI:** 10.1136/bmjopen-2016-012505

**Published:** 2016-12-23

**Authors:** Paul Henry Yerrell, David Roder, Margaret Cargo, Rachel Reilly, David Banham, Jasmine May Micklem, Kim Morey, Harold Bundamurra Stewart, Janet Stajic, Michael Norris, Alex Brown

**Affiliations:** 1 Wardliparingga Aboriginal Research Unit, South Australian Health and Medical Research Institute, Adelaide, South Australia, Australia; 2 Centre for Population Health Research, University of South Australia, South Australian Health and Medical Research Institute, Adelaide, South Australia, Australia; 3 Cancer Epidemiology Group, Centre for Population Health Research, University of South Australia, South Australian Health and Medical Research Institute, Adelaide, South Australia, Australia

**Keywords:** Indigenous Health, ONCOLOGY, QUALITATIVE RESEARCH, Data linkage

## Abstract

**Introduction:**

In Australia, Aboriginal and Torres Strait Islander People carry a greater burden of cancer-related mortality than non-Aboriginal Australians. The Cancer Data and Aboriginal Disparities Project aims to develop and test an integrated, comprehensive cancer monitoring and surveillance system capable of incorporating epidemiological and narrative data to address disparities and advocate for clinical system change.

**Methods and analysis:**

The Advanced Cancer Data System will integrate routinely collected unit record data from the South Australian Population Cancer Registry and a range of other data sources for a retrospective cohort of indigenous people with cancers diagnosed from 1990 to 2010. A randomly drawn non-Aboriginal cohort will be matched by primary cancer site, sex, age and year at diagnosis. Cross-tabulations and regression analyses will examine the extent to which demographic attributes, cancer stage and survival vary between the cohorts. Narratives from Aboriginal people with cancer, their families, carers and service providers will be collected and analysed using patient pathway mapping and thematic analysis. Statements from the narratives will structure both a concept mapping process of rating, sorting and prioritising issues, focusing on issues of importance and feasibility, and the development of a real-time Aboriginal Cancer Measure of Experience for ongoing linkage with epidemiological data in the Advanced Cancer Data System. Aboriginal Community engagement underpins this Project.

**Ethics and dissemination:**

The research has been approved by relevant local and national ethics committees. Findings will be disseminated in local and international peer-reviewed journals and conference presentations. In addition, the research will provide data for knowledge translation activities across the partner organisations and feed directly into the Statewide Cancer Control Plan. It will provide a mechanism for monitoring and evaluating the implementation of the recommendations in these documents.

**Strengths and limitations of this study**

- This mixed-methods study:
- Addresses significant gaps in the quality and comprehensiveness of cancer data in South Australia, with a particular focus on cancer among Aboriginal and Torres Strait Islander people.
- Aims to link epidemiological and experiential data in a unique and sustainable Advanced Cancer Data System for continuous quality improvement of cancer care for Aboriginal and Torres Strait Islander people.
- Is underpinned by principles of community engagement and participation to ensure relevance and utility for the Aboriginal and Torres Strait Islander Community.
- Limitations include:
- A reliance on the willingness of data custodians to release data for inclusion in the Advanced Cancer Data System.
- Difficulty in reaching those Aboriginal and Torres Strait Islander people who do not take up standard medical care, due to recruitment occurring through hospitals and health services. This will be mitigated by including the service providers and family members as participants to provide a broader view of cancer experiences in Aboriginal communities.

## Introduction

Nationally, Aboriginal and Torres Strait Islander Australians (hereafter ‘Aboriginal people’) carry a significantly greater burden of cancer mortality than the general population, despite an equivalent or slightly lower cancer incidence.^1^ Aboriginal people entering the health system for cancer treatment tend to be younger, have more advanced cancer and more lethal types of cancers than non-Aboriginal Australians.^2^ ^3^ The drivers of this disparity are varied, relating to a higher rate of exposure to risk factors including but not limited to smoking, lower uptake of cancer screening and higher rates of comorbidity.^4^ There is also evidence that once diagnosed, Aboriginal people are less likely than other Australians to receive comprehensive and complete cancer treatment.^5^ ^6^ While the non-Aboriginal community has experienced improvement in cancer outcomes, the same improvement has not been observed in the Aboriginal community, resulting in a widening of the disparity between Aboriginal and non-Aboriginal Australians in relation to cancer mortality.^2^ ^7^

Australia has mandatory reporting requirements for invasive cancers to registries, with the exception of non-melanoma skin cancers. State and territory population-based cancer registries receive information from a variety of sources including hospitals, pathology laboratories, radiotherapy centres and registries of Births, Deaths and Marriages (BDM).^8^ For Aboriginal people, registry data collection is hampered by an inaccurate and incomplete recording of Aboriginal status, resulting in inaccuracies in comparisons between states and territories and assessments of national secular trends, cancer burden, incidence and survival.^9–11^ Few Australian cancer registries routinely record diagnostic stage, which hampers the ability to adequately interpret comparative survival outcomes for Aboriginal people and non-Aboriginal Australians.^9^ ^10^ Nationally, no registries routinely record comorbidity—a critical deficiency given that comorbidity can significantly influence the choice and prescription of chemotherapy and other cancer therapies, and cancer outcomes.^10^ ^12^ Treatment data have also not been collected routinely by registries.

To overcome these deficits, data linkage has been used in some states in Australia to combine cancer registry and treatment data.^5^ ^9^ ^13–17^ These linkage studies have demonstrated the value of assessing cancer outcomes in relation to patient treatment, comorbidity and various sociodemographic features. Work in New South Wales has compared survival and surgical treatment of Aboriginal and other Australians with breast, colorectal, non-small cell lung and prostate cancers by linking their cancer registry records with hospital admission and death records.^13^ ^15–17^ However, this practice is not yet incorporated into most routine registry data collection processes in Australia.

In regard to the experiences of Aboriginal people with cancer, studies have identified barriers to care relating to transport, the hospital environment, separation from family and country, racism and potentially dangerous misunderstandings through language and cultural differences.^6^ ^14^ ^18^ ^19^ However, these types of data are not collected routinely for the purpose of healthcare quality improvement. Given that healthcare reform is best guided by the experience of those needing and seeking its support, the omission of data on Aboriginal experiences of cancer care represents a significant gap in the range of data currently collected. The views and experiences of service providers, although frequently overlooked, are also critical in focusing on structural and patient-related issues for reform.

To address these gaps, the Cancer Data and Aboriginal Disparities (CanDAD) project will develop and test an integrated, comprehensive cancer monitoring and surveillance system for Aboriginal people in South Australia, which is likely to have relevance to other regions. This Advanced Cancer Data System (ACaDs) will be developed explicitly with Aboriginal people, to identify prevention strategies and improve the quality of cancer care provided to Aboriginal people.

The specific objectives of CanDAD, across three distinct phases of the research project, include:

### Phase 1: Improving the quality and completeness of South Australian cancer data

1. To ensure accurate and comprehensive recording of data for Aboriginal and non-Aboriginal people in South Australia across a range of cancer, cancer screening, treatment, diagnostic and health service indicators;
2. To establish methods for accurate, complete and sustainable ongoing monitoring of cancer by type of cancer, mode of detection and treatment, and for monitoring outcomes among Aboriginal patients with cancer;
3. To assess disparities between Aboriginal and non-Aboriginal South Australians in incidence, mortality, survival, stage, stage-adjusted survival, extent of comorbidity and technical appropriateness of treatment received, by sociodemographic strata such as geographic remoteness.

### Phase 2: Exploring experiences of cancer care

1. To develop a comprehensive understanding of patient and provider perspectives on service access, barriers and enablers to care, service quality, acceptability and appropriateness;
2. To develop a brief, culturally sensitive self-report instrument for recording and quantifying satisfaction of Aboriginal patients with cancer with system performance that can be deployed as part of routine service delivery;
3. To prioritise service improvements to enhance Aboriginal people's cancer experiences.

### Phase 3: Towards an ACaDs

1. To develop a streamlined, integrated data system and linkage infrastructure for ongoing, timely monitoring of cancer diagnoses, services and outcomes for guiding health policy;
2. To explore the potential for automated cancer data collation for South Australia into the future and to collaboratively plan its implementation with partner organisations.

## Methods and analysis

**Figure 1:**
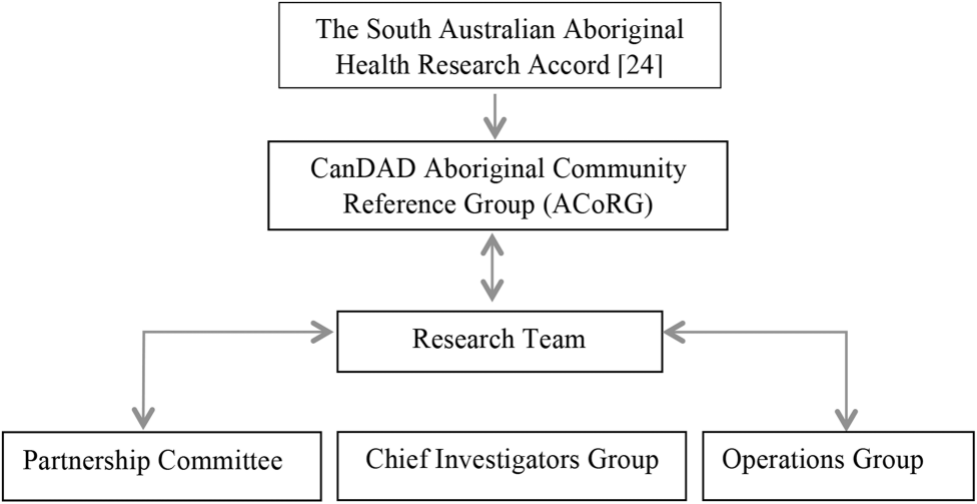
Governance Structure of Cancer Data and Aboriginal Disparities (CanDAD), following the South Australian Aboriginal Research Accord.

The Aboriginal Community Reference Group (ACoRG) is playing a key role in ensuring that methodological processes are culturally appropriate and aligned with Aboriginal community priorities (figure 1). The six members, both female and male, representing different remote, regional and urban locations across South Australia, are Elders and cancer survivors with a commitment to doing research *the* ‘right way,’ as articulated in the South Australian Aboriginal Health Research Accord,^20^ and raising the Community's role in changing cancer services. Through regular meetings, the group will have an opportunity to interpret and translate epidemiological and narrative data through Aboriginal cultural lenses.

### Phase 1: Improving the quality and completeness of SA cancer data

Extending work already undertaken during the pilot phase of the project, the quality and completeness of data identifying Aboriginal status in the South Australian (SA) Cancer Registry will be improved by cross-matching against records from SA Health's inpatient hospital collection, death registrations and the South Australia-Northern Territory DataLink's existing SA Master Linkage File. Where any records indicate that the person is Aboriginal, they will be included under broad, inclusive case criteria. The validity of each case will then be reviewed for retention and subsequent sensitivity analysis using more stringent criteria such as country of birth and family name. Aboriginal people living in South Australia at the time of their cancer diagnosis between 1990 and 2010 are estimated to number around 1000 and will be used for methodological Research and development (R&D) and contribute baseline data for the ACaDS being developed. Where possible, each cohort member will be matched to a non-Aboriginal person on the basis of: (a) year of birth; (b) sex; (c) year of diagnosis; and (d) cancer type (primary organ site). A single, randomly selected member will be included where there are multiple candidates for the non-Aboriginal cohort. Following this R&D, these initial data will be used to decide on numbers of non-Aboriginal people to optimise statistical power in the prospective data system. Each cohort member's diagnosed cancer will then be manually staged by the SA Cancer Registry staff using the Surveillance, Epidemiology, and End Results (SEER) Program summary stage criteria as an indicator of the extent of spread of cancer from its point of origin.

**Figure 2:**
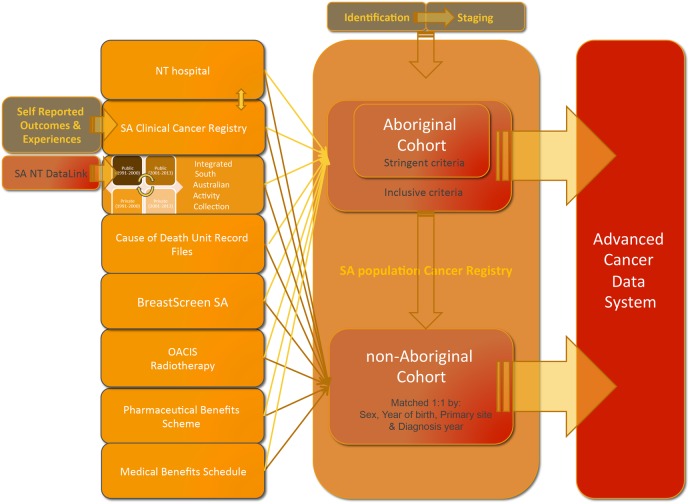
Outline of the process from de-identified service and patient outcome data to cohort members to ACaDs. ACaDs, Advanced Cancer Data System; NT, Northern Territory; OACIS, Open Architecture Clinical Information System; SA, South Australia.

In addition to the patient identifier administered by the SA Cancer Registry, each cohort member will be assigned a unique and randomly generated project linkage key, which will attach to any clinical or administrative record belonging to that individual across all of the data sets sourced (figure 2). The use of linkage keys removes the need for person identified data to be supplied to, or stored in, the ACaDS integrated data set. These protocols employ a combination of probabilistic (linking) and deterministic (merging) techniques to achieve the highest possible quality of record integration between these data sets.

Each data set has unique characteristics and ACaDS integration processes need to be tailored to maximise the contribution of each to project goals. For example, the Integrated South Australian Activity Collection (ISAAC) contains information about inpatient separations from public and private hospitals in South Australia. These records are held in four series: public and private hospital records from the 1990s, and post-2000. All four series are available to ACaDS in a de-identified form stripped of names and addresses but maintaining a hospital-specific patient unit record number (URN), sex, date of birth and residential area location(s). This enables a consistent, ‘bronze’ standard integration approach^21^ for interconnecting an individual's records across hospitals and connecting back to the health service and URN recorded on the SA Cancer Registry (operational protocol details are available from the authors on request). Identified data are available to SA-NT DataLink for conducting gold standard integration of contemporary public hospital records with the SA Cancer Registry. The results of this linkage are also available to ACaDS and provide an important means of assessing the quality of the bronze standard approach with historic records while facilitating ongoing intelligence on the hospital-specific URNs associated with people diagnosed with cancer into the future. The end result for ACaDS will be the inclusion of valuable material on comorbid conditions as well as the treatment and procedures (cancer and otherwise) experienced by cohort members.

The remaining South Australian data collections will make other unique contributions to ACaDS. For instance, when matched to the SA Cancer Registry using registration numbers from the BDM data collection, the Cause of Death Unit Record Files will provide International Classification of Diseases (ICD) coded causes of death for non-cancer deaths. This will be the first use of these data in this way in South Australia, and will add to the descriptive and interpretative power of registry data into the future. Also, the Open Architecture Clinical Information System (OACIS) Radiotherapy data set will be used to validate and complement data on radiotherapy obtained from the SA Cancer Registry, ISAAC and national health insurance data. Other data sets held nationally also have great potential for informing ACaDS. For example, cohort members’ health insurance data from the Pharmaceutical Benefits Scheme (PBS) and Medical Benefits Schedule (MBS) will help enumerate critical issues of: chemotherapy uptake; comorbid disease management in primary care; and actual compared with recommended treatment pathways.

The process is for data custodians to supply de-identified South Australian data with project linkage keys directly to an ACaDS secure data storage environment hosted within the South Australian Health and Medical Research Institute (SAHMRI) and University of South Australia. The linkage keys will be used to merge or ‘integrate’ each cohort member's clinical and administrative records. They may be used to incorporate any de-identified patient-reported experience data gathered under phase II or later, which could be held as a field on the linked data set, for instance. This best practice method of data integration will inform analysis of cancer types, stage, other cancer prognostic characteristics, comorbidity, clinical management, patterns of care, health system characteristics (including estimated travelling distances to treatment centres) and, for each Aboriginal cohort member, patient reported and where possible provider, family and carer reported experience (table 1). Commonwealth data will be integrated with South Australian data and stored for remote data analysis in the Secure Unified Research Environment (SURE).^22^

**Table 1:** De-identified data variables to be included in ACaDS

Category	Variables
Demographics	Age, gender, Aboriginal and Torres Strait Islander status, country of birth, postcode or other location of residence at diagnosis, residential remoteness and residential-area based measure of socioeconomic status
Cancer diagnosis	Cancer screening histories (for breast, cervix, once the HPV screening register is available, and bowel cancers), clinical basis of cancer diagnosis, date of diagnosis, primary organ site and morphology (ICD coded), histopathology grade at diagnosis, breast cancer size (mm/nodal status/focality), and potentially melanoma thickness and level (note: melanomas will be rare)
Stage at diagnosis	SEER summary stage (expressed as local, regional or distant degree of spread of solid tumours) and, where possible, Registry derived TNM stage (derived from pathology forms, hospital narrative reports and case notes)
Treatment	Surgery, surgery type (ACHI codes), surgery date, timing of radiotherapy initiation, chemotherapy and other systemic therapy start date, agent type (where available) and any other recorded treatments (used to establish treatment patterns and completeness)
Death	Date, cause (ICD coded) and place (major metropolitan public hospital, other public hospital, private hospital, aged care facility, hospice and home/private residence, extracted by SA Cancer Registry staff from official death registrations)
Comorbidity	ICD coded major ICD disease chapter; comorbidity index (Charlson/other)—primarily derived from public and private hospital coding, public hospital notes, MBS and PBS claims, and death records

ACaDs, Advanced Cancer Data System; ACHI, Australian Classification of Health Interventions; HPV, human papillomavirus; ICD, International Classification of Diseases; MBS, Medical Benefits Schedule; PBS, Pharmaceutical Benefits Scheme; SA, South Australia; SEER, Surveillance, Epidemiology, and End Results; TNM, tumour-node-metastasis.

These data will be used to quantify differences between Aboriginal and non-Aboriginal Australians with cancer, regarding: basis of diagnosis; cancer stage at diagnosis, histopathology grade and other prognostic characteristics; extent and type of comorbidity; unadjusted and adjusted survival (adjusted for stage, grade, other prognostic characteristics and comorbidity); treatment types and technical appropriateness; and residential area derived remoteness (Australian Standard Geographical Classification index), socioeconomic status (Socioeconomic Indexes for Areas) and other sociodemographic descriptors. The statistical power will be the maximum power that these numbers provide. This will be dependent on the numbers of Aboriginal people with cancer and the numbers of non-Aboriginal people chosen for comparison.

SA Cancer Registry records augmented with the SEER summary stage at diagnosis and causes of non-cancer death will be analysed to address Aboriginal community questions. Specifically, Aboriginal people are interested in knowing why Aboriginal patients with cancer are more likely to die prematurely than non-Aboriginal patients. Where they die of non-cancer-related causes, they are interested in knowing which causes contributed. Analyses also will address the prevalence of comorbid conditions and their association with survival outcomes and patterns of care. Other health and social data sets already have linkage keys assigned through the SA-NT DataLink (the SA Master Linkage File) and may allow ACaDS to describe and quantify broader determinants of cancer diagnosis, treatment success and survivorship, including educational, housing, disability and mental health characteristics.

### Phase 2: Exploring experiences of cancer care

In phase 2, qualitative work will involve the collection of stories from Aboriginal people with experience of cancer; family members and carers; as well as service providers working with Aboriginal people with cancer, in urban, regional and remote locations. This will form the foundation of a participatory process of questionnaire development, enabling the inclusion of experiential data in the Advanced Cancer Data Monitoring System (ACaDS).^23^ The stakeholders involved in this process will include Aboriginal community members, alongside representatives from governmental and non-governmental agencies engaged in providing cancer services. A concept-mapping process will occur in concert with the development of a brief Aboriginal Cancer Measure of Experience (ACME) instrument for recording and quantifying satisfaction of Aboriginal patients with cancer with system performance, thus contributing to ACaDS.

The specific research questions to be addressed in phase 2 are:

1. What are the barriers and enablers of access, quality and continuity of care for Aboriginal people with cancer, as identified by Aboriginal people themselves, their families, carers and service providers?
2. When interacting with the health system, what are the concerns and priorities of Aboriginal people with cancer, their families, carers and service providers?
3. What constitutes high quality, acceptable and appropriate care for Aboriginal people with cancer?

#### Data collection

Participants will be recruited through Aboriginal Cancer Care Coordinators at a major metropolitan hospital and from Aboriginal Community Controlled Health Services in a mix of purposive and snowball sampling. Care will be taken to make the sample as broadly representative as possible of the geographically and culturally diverse Aboriginal populations within South Australia, and with regard to age, gender and cancer type. Those who travel to South Australia for treatment from interstate, as routinely occurs for patients from the Northern Territory, will be included in the sample. Based on discussions with the Aboriginal Health Research Ethics Committee, and following a brief literature review on ‘timing to inform recruitment protocols and the conduct of the interview’, sensitivity will be shown regarding appropriateness of approaches for contacting patients with cancer at different phases of treatment. Given the particular emotional factors arising between time of diagnosis and treatment, participants will not be approached during that period. Furthermore, with the varying timelines of individual clinical events, recruitment may mean approaching patients at various points postdiagnosis.^24–28^ Inclusion of participants will cease at the point of relative data saturation and when researchers and the ACoRG reach consensus that, as far as practically possible, the sample is representative in relation to the categories noted above.

With a view to enabling a culturally safe environment, participants will be invited to choose between a male, female, Aboriginal or non-Aboriginal interviewer and to nominate their preferred interview location. The qualitative (narrative) component of the CanDAD project is grounded in concepts drawn from participatory action research and Aboriginal methodologies which move away from the positivist paradigm towards those that more closely resemble Aboriginal terms of reference.^29^ ^30^ The important role of storytelling, or yarning, in Aboriginal cultures will be honoured by initially providing participants the time and space to tell their story in their own words, with their own emphasis.^31^ ^32^ In this way, the methods move away from defining needs and outcomes in terms of established biomedical or functional terms, and towards descriptions that are relevant to the contexts of Aboriginal communities and life histories.^33^ Interviews will be audiorecorded, transcribed verbatim and returned to participants for checking if requested. Transcripts will be de-identified prior to analysis.

#### Data analysis

**Figure 3:**
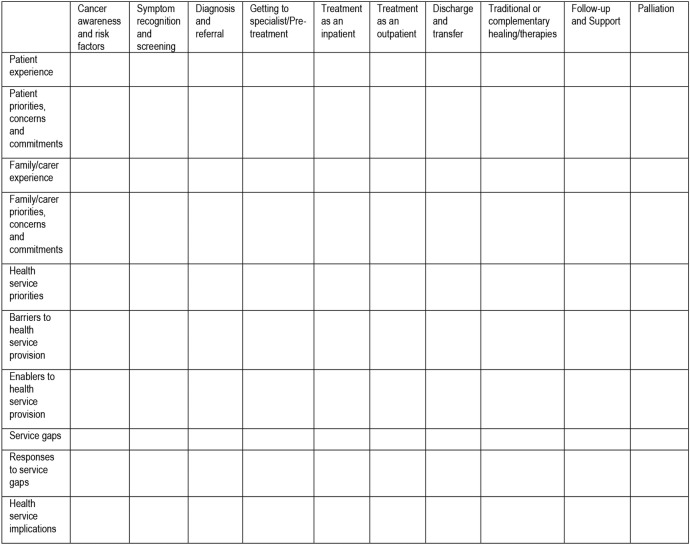
Cancer pathway mapping tool.

Patient journey mapping has been used in various ways to guide health system review, and to support integrated and patient-centred care in situations where patients interact with multiple providers in different settings over extended periods of time.^34–36^ For CanDAD, mapping tools developed for use with Aboriginal patients^37^ ^38^ will be adapted to reflect the stages of a cancer journey as outlined in the Statewide Cancer Control Plan^39^ and incorporating elements from the Aboriginal and Torres Strait Islander Companion Document to this plan,^40^ as shown in figure 3. Patient journey mapping enables stories to be analysed from multiple perspectives, or according to their component parts, while also maintaining and honouring the narrative as a coherent whole. This is important in the light of concerns about Western reductionism that can work against indigenous research priorities.^29^ ^41^ Since the term ‘cancer journey’ was not preferred by the ACoRG, the term ‘patient pathway mapping’ has been adopted. Within the Statewide Cancer Control Plan, there are several classifiable circumstances that occur in the prediagnosis, treatment and post-treatment phases of cancer patient pathways. However, individual factors such as demographic factors, patient preferences, access to services and type of cancer determine if and when these circumstances occur.

Following the methodology used by Graneheim and Lundman,^42^ the transcribed text will be divided into meaning units (categories) reflecting the manifest content of the data, which will be mapped onto the patient pathway tool (see figure 3). Steps in the pathway (columns) will be analysed across multiple participant narratives so that dominant themes are identified at each stage or across stages. Subgroup analysis by gender, residence (urban, regional, remote), age and cancer type will be conducted for patients, survivors, family/carers and service providers. Health service priorities outlined the Statewide Cancer Control Plan and the National Aboriginal and Torres Strait Islander Cancer Framework 2015 will be identified and compared with patient and family/carer priorities within and across narratives.

Underlying themes that emerge across the patient pathway will also be identified and described using language that closely reflects that used by the participants,^42–44^ as well as that reflecting Aboriginal understandings of health and well-being.^45^ ^46^ In this way, factors that may be important influences on the patient pathway, but do not fit neatly into a particular stage, will be captured. Examples may include deeply personal psychosocial aspects of cancer pathways such as connectedness to Culture, Community and Country, family support, or reflections on maintaining well-being in the face of cancer. Member checking with a subgroup of interviewees will occur prior to the last round of interviews, alongside peer de-briefing. The ACoRG will also provide specific attention to the interpretation of data. At the completion of stage 1, findings from the patient pathway and thematic analysis will be presented to a stakeholder workshop convened for the purpose of refining the priorities that will drive the concept-mapping and self-report instrument development outlined below.

#### Concept mapping

Concept mapping^23^ is a participatory planning tool that is used to identify service delivery priorities based on perceptions of Aboriginal people affected by cancer and cancer service providers. Concept mapping is guided by a ‘prompt’ question (eg, ‘What action needs to be taken to improve the quality of Aboriginal patients’ pathway in the primary healthcare and hospital systems?’). In this study, the prompt question will be generated by the Operations Group, ACoRG and project investigators. The initial pool of strategies for improving the quality of Aboriginal cancer pathways will be identified from the qualitative analysis (in the form of statements) and refined during the workshop aforementioned.

Following the process outlined by Kane and Trochim,^47^ a final pool of ∼80 strategies will be sorted and rated on their perceived importance and feasibility of implementation in the primary healthcare and hospital systems. Ratings will be analysed using multidimensional scaling, hierarchical cluster analysis and bridging analysis. Pattern matching will provide information on how to target intervention strategies to geographic location (ie, rural, remote, metro) and the system's level (ie, individual, family, community, primary healthcare, hospital). Members from the Operations Group and the ACoRG will be actively engaged in interpreting and translating the results into meaningful local and statewide actions to improve the quality of Aboriginal cancer pathways.

#### Development of the ACME

The concept mapping and development of the ACME will proceed in parallel, to maximise the relevance and utility of the self-report instrument while avoiding overburdening stakeholders. Since the content and format of the ACME will be guided by the findings and the participatory process of development, it is not possible to be prescriptive about its content at this stage. The development process will follow Streiner and Norman's^48^ procedures for developing instruments with face validity, content validity and reliability, and will be informed by the growing literature on patient-recorded outcome and experience measures and quality of life measurement.^49^ ^50^ Domains in the ACME will be identified on the basis of the patient pathway mapping and thematic analysis. The barriers and enablers to care and underlying themes will be used to generate item-level statements within each identified domain. The ACME will be pilot-tested and refined initially with the involvement of the ACoRG, then within Aboriginal primary healthcare settings and finally by the Aboriginal Cancer Care Coordinators in the tertiary setting.

### Phase 3: Towards an ACaDS

Phase 3 seeks to embed these data sources and methods into routine cancer data collection and collation, using data linkage of cancer registry, other routinely collected data extracts and a service-level recording of self-reported patient experience of care. These data will be collated and provide the substrate for extensive partner feedback and participatory cycles with governance committees to explore and interpret the findings. Through ongoing engagement with cancer service providers, Aboriginal people and organisations, the partnership will provide data to assess, test and modify ACaDS progressively, so that it retains currency and is of high quality and adaptive to changing needs. ACaDS is expandable into the future. Additional health and social data sets will be assessed for relevance to CanDAD's future and ongoing aims, as well as efficiency and sustainability requirements. Routine standard analyses of monitoring system data and presentation of results will be constructed in an attractive/readily interpretable form for different audiences. Our participatory methods and partner engagement will be directed at efficiently sustaining the system, data collation, collection and usage and governance processes into the future.

## Ethics and dissemination

The Australian Institute of Health and Welfare (AIHW) Human Research Ethics Committee (HREC) approved a proposal to incorporate MBS and PBS data into ACaDS. The Central Australian Health Research Ethics Committee (CAHREC) has been approached to approve the integration of Northern Territory hospital records of South Australians experiencing cancer diagnoses and hospitalisation in that territory. The data linkage processes will comply with the privacy principles established by the Population Health Research Network (PHRN). In addition, operational protocols developed with each data custodian have been provided to SA Health HREC. All participants will provide written informed consent for participation in study interviews.

Findings will be disseminated in local and international peer-reviewed journals. Proposed research methods and preliminary findings have been discussed at local and international conferences^51–57^ and in an invited editorial.^58^ In addition, CanDAD is providing data for knowledge translation activities across the partner organisations, including direct input into the Statewide Cancer Control Plan and the Aboriginal and Torres Strait Islander Companion Document.^40^ It will provide a mechanism for monitoring and evaluating the implementation of the recommendations in these documents.
